# Paxillin regulates liver fibrosis via actin polymerization and ERK activation in hepatic stellate cells

**DOI:** 10.1242/jcs.261122

**Published:** 2023-09-28

**Authors:** Nour Hijazi, Zengdun Shi, Don C. Rockey

**Affiliations:** Digestive Disease Research Center Core, Medical University of South Carolina, Charleston, SC 29425, USA

**Keywords:** Adhesion, Cytoskeleton, Focal adhesions, Cirrhosis

## Abstract

Liver injury leads to fibrosis and cirrhosis. The primary mechanism underlying the fibrogenic response is the activation of hepatic stellate cells (HSCs), which are ‘quiescent’ in normal liver but become ‘activated’ after injury by transdifferentiating into extracellular matrix (ECM)-secreting myofibroblasts. Given that integrins are important in HSC activation and fibrogenesis, we hypothesized that paxillin, a key downstream effector in integrin signaling, might be critical in the fibrosis pathway. Using a cell-culture-based model of HSC activation and *in vivo* models of liver injury, we found that paxillin is upregulated in activated HSCs and fibrotic livers. Overexpression of paxillin (both *in vitro* and *in vivo*) led to increased ECM protein expression, and depletion of paxillin in a novel conditional mouse injury model reduced fibrosis. The mechanism by which paxillin mediated this effect appeared to be through the actin cytoskeleton, which signals to the ERK pathway and induces ECM protein production. These data highlight a novel role for paxillin in HSC biology and fibrosis.

## Teaser

**Highlighted Article:** Paxillin is shown to be a key molecule important in the function of fibrosis effector cells, which emphasizes a novel role for it in liver fibrosis and cirrhosis, a leading cause of morbidity and mortality worldwide.

## INTRODUCTION

Chronic liver diseases account for around two million deaths per year worldwide (Asrani et al., 2019). Exposure of the liver to certain agents (e.g. ethanol, viral hepatitis and others) injures hepatocytes (the primary epithelial cells in the liver, making up almost 80% of all liver cells), triggering an inflammatory wound healing response (Malhi and Gores, 2008; Stanger, 2015). This response leads to the activation of effector cells, which produce excessive extracellular matrix (ECM) material (Pinzani, 2015). Aberrant ECM production associated with this wounding response eventually causes extensive scarring or fibrosis, stiffening and organ dysfunction (Rockey et al., 2015). It has now been well established that the primary effector cell responsible for aberrant ECM production in liver fibrosis is the perisinusoidal hepatic stellate cell or HSC (Higashi et al., 2017; Mederacke et al., 2013). Of mesenchymal origin, HSCs are ‘quiescent’ in normal liver, but after injury, become ‘activated’ by transdifferentiating into ECM-secreting myofibroblasts that are also proliferative, migratory, contractile and resistant to apoptosis (Puche et al., 2013). As the primary source of ECM after liver injury, understanding their biology is essential in order to identify novel potential therapeutic targets.

Mechanotransduction is the process by which cells sense and respond to their physical microenvironment. Paxillin, a multidomain adapter protein, is one of the many proteins implicated in the cellular response to mechanical forces from the ECM (Jansen et al., 2017). Paxillin localizes to focal adhesions – dynamic multi-protein structures that form at sites of cell contact with the ECM (Hynes, 2002). It harbors five LD motifs, four LIM domains, an SH2 domain-binding site, and an SH3 domain-binding site (Schaller, 2001). These structures allow paxillin to serve as a platform for the recruitment of many regulatory and structural proteins, and to mediate downstream signaling (Deakin and Turner, 2008). Paxillin has also been shown to link the ECM to the actin cytoskeleton inducing its reorganization, a process necessary for cellular functions in wound healing, such as ECM synthesis (López-Colomé et al., 2017; Turner, 2000).

Work from our group and others has demonstrated the importance of actin dynamics in HSC biology. The development of a robust actin cytoskeleton is a characteristic of HSC activation and differentiation into the myofibroblast phenotype, and is important in HSC functions such as cell migration, cell adhesion and attachment, and cellular contractility. The actin cytoskeleton also plays a role in ECM production (particularly type I collagen; also known as Col I) via the ERK pathway, a major pathway involved in fibrogenesis (Shi and Rockey, 2017; Rockey et al., 2013, 2019).

Here, we tested the hypothesis that paxillin plays a key role in hepatic fibrogenesis by regulating type I collagen production during stellate cell activation via the actin-ERK pathway. We utilized both *in vitro* and *in vivo* models of liver injury with primary HSCs to show that paxillin is upregulated in activated HSCs and in the injured liver. Moreover, overexpression of paxillin in HSCs stimulated their activation and increased type I collagen expression. Blocking ERK1 and 2 (ERK1/2; also known as MAPK3 and MAPK1, respectively) activation and actin polymerization abrogated the increase in type I collagen associated with paxillin overexpression. Finally, overexpression of paxillin *in vivo* stimulated liver fibrosis, and its deletion from HSCs in a novel conditional knockout mouse decreased liver fibrosis. Our results suggest that paxillin plays an important role in the fibrogenic response to liver injury and highlight a potential novel therapeutic pathway.

## RESULTS

### Paxillin expression is upregulated following *in vitro* and *in vivo* HSC activation

**Fig. 1.**
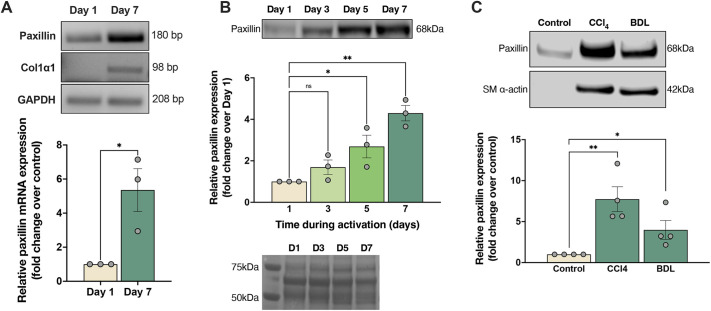
**Paxillin expression increases during HSC activation.** (A) HSCs were isolated from normal rat liver HSCs and allowed to undergo culture-induced activation. Total RNA was isolated, and cDNA was generated as described in the Materials and Methods. A representative agarose gel electrophoresis image showing PCR products of paxillin, *Col1a1* (col1α1; type I collagen, α1 chain) and the *Gapdh* gene is shown. Paxillin mRNA expression was detected by qPCR and quantitative data are shown below (*n*=3 biological replicates). (B) HSCs were isolated as above, and cell lysates (30 µg total per sample) were subjected to immunoblotting to detect paxillin as described in the Materials and Methods. A representative immunoblot is shown at the top. Bands were scanned and quantified by normalization to the level of control cell expression (day 1) (*n*=3 biological replicates). A Ponceau S-stained membrane to show equal protein loading is shown below the graph. (C) Liver injury was induced by administration of six doses of CCl_4_ and by BDL as described in the Materials and Methods. HSCs were isolated from normal (serving as control) and fibrotic rat livers 5 days after the final gavage for the CCl_4_ model and 14 days after ligation of the bile duct for the BDL model, and paxillin was detected by immunoblotting (SM, smooth muscle). Bands were scanned and quantified by normalization to the level of control cell expression (HSCs from normal liver) (*n*=4 biological replicates). Error bars represent the mean±s.e.m. *P<0.05; **P<0.01; ns, not significant two-tailed unpaired Student's *t*-test in A, one-way ANOVA with Tukey multiple comparisons in B and C

Given the putative involvement of focal adhesions and integrins in the fibrogenic response, we examined the expression of the prominent focal adhesion molecule paxillin in HSC activation. In a primary cell culture model that closely recapitulates *in vivo* HSC activation (Iredale, 2007), paxillin expression increased dramatically during activation both at the mRNA and protein levels (Fig. 1A and B, respectively). Ponceau S staining was used to show equal protein loading (Fig. 1B, lower panel; Fig. S1A).

We next examined the expression of paxillin in two *in vivo* liver injury models – carbon tetrachloride (CCl_4_) and bile duct ligation (BDL) (Yanguas et al., 2016). CCl_4_ administered by gastric gavage is metabolized by the liver into a radical that binds to cellular molecules (nucleic acids, proteins and lipids), and initiates a lipid peroxidation chain reaction resulting in hepatocellular injury, inflammation and eventually fibrosis (Weber et al., 2003). On the other hand, surgically ligating the bile duct leads to the expulsion of toxic bile acids in the liver, which causes hepatocyte necrosis and inflammation (Szabo et al., 2015). Consistent with the robust *in vitro* cell culture model of activation, paxillin expression in activated HSCs isolated from fibrotic livers was significantly upregulated compared to HSCs isolated from normal livers (Fig. 1C). Ponceau S staining was used to show equal protein loading (Fig. S1B). Collectively, these data indicate that paxillin is upregulated in HSCs during the fibrogenic response.

### Paxillin-containing focal adhesions colocalize with the actin cytoskeleton in HSCs

**Fig. 2.**
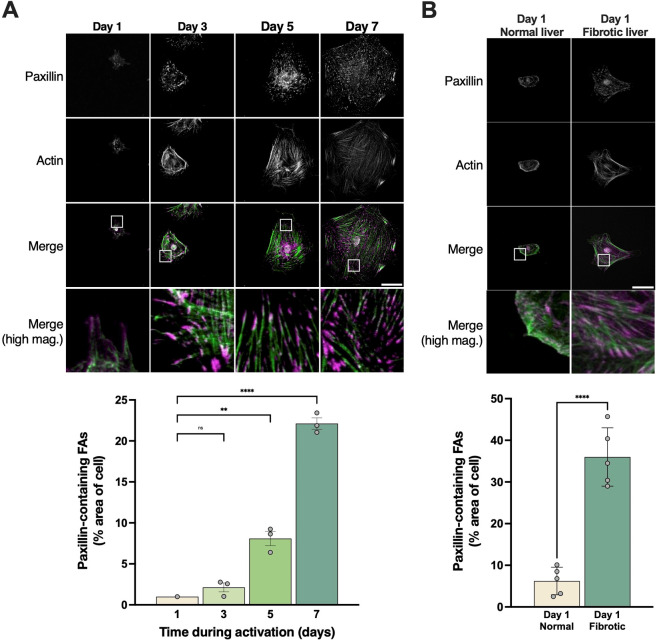
**Paxillin localization during HSC activation.** (A) HSCs were isolated, grown on glass coverslips for 7 days, and fixed. Paxillin was labeled at the specified time points as described in the Materials and Methods. DAPI was used to label nuclei. The boxes in row 3 of the panel illustrate magnified area in the fourth row of the panel. Scale bar: 30 μm. Paxillin-containing FAs were quantified as described in the Materials and Methods and depicted in the graph below the image panel (note that each data point represents one biological replicate). (B) HSCs were isolated from normal livers and from fibrotic livers (CCl_4_ model) and grown on glass coverslips overnight. Cells were fixed and paxillin was labeled as in A. Images shown are representative of at least 10 others. Paxillin-containing FAs were quantified as in A (*n*=5 technical replicates). Scale bar: 10 μm. Error bars represent the mean±s.e.m*.* **P<0.01; ****P<0.0001; ns, not significant (one-way ANOVA with Tukey multiple comparisons in A, two-tailed unpaired Student's *t*-test in B).

Given that activated HSCs exhibit both robust paxillin expression (Fig. 1) and a prominent actin cytoskeleton (Shi et al., 2020), we hypothesized that in the activation process, the area of paxillin-containing FAs would increase, and the paxillin-containing FAs would be tightly associated with actin stress fibers. As expected, during culture-induced activation, paxillin-containing FAs were prominently distributed throughout the cell, particularly at the ends of actin filaments (Fig. 2A). When HSCs were fully activated (day 7), paxillin-containing FAs occupied ∼20% of the cell area (Fig. 2A, bottom panel). *In vivo* activated HSCs isolated from fibrotic mouse livers exhibited paxillin expression in a pattern identical to culture-activated HSCs (Fig. 2B). The percentage area of the cell occupied by paxillin-containing FAs was determined as previously described (Horzum et al., 2014) and explained in the Materials and Methods section. These data further support the postulate that paxillin expression is elevated in activated HSCs, and suggest that paxillin-containing FAs dynamically change throughout the process of activation.

### Paxillin regulates HSC migration, attachment and proliferation

**Fig. 3.**
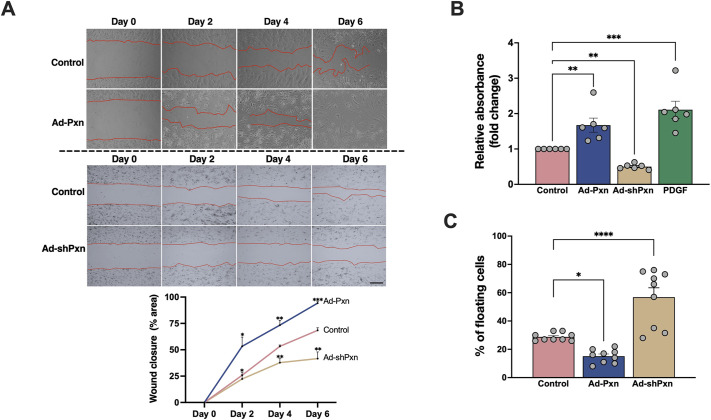
**Functional effects of paxillin in HSCs.** (A) HSCs were isolated and after 1 day in culture, exposed to Ad-Pxn, Ad-shPxn and a control adenovirus for 2 days. Subsequently, a scratch was made using a p20 pipette tip and wound closure was assessed by measuring the ratio of wound area at the indicated time points to the original gap at *t*=0 (*n*=3 biological replicates). Representative images and a graph depicting quantification of cell migration are shown. Scale bar: 100 μm. (B) HSCs were grown for 2 days and then exposed to adenoviruses as in A or 10 ng/μl PDGF (which was used as a positive control for proliferation). Proliferation was measured as described in the Materials and Methods (*n*=6 technical replicates from three biological replicates). (C) HSCs were cultured as in B, and a cell detachment assay was performed as described in the Materials and Methods (each data point represents the percentage of floating cells divided by floating and adherent cells as described in the Materials and Methods; *n*=9 technical replicates from three biological replicates). Error bars represent the mean±s.e.m*.* *P<0.05; **P<0.01; ***P<0.001; ****P<0.0001 (one-way ANOVA with Tukey multiple comparisons). Ad, adenovirus; Pxn, paxillin.

To further understand the functional effects of paxillin in HSCs, we modulated paxillin expression and determined subsequent effects on phenotypes associated with HSC activation (migration, attachment and proliferation). We created an adenovirus that harbors a full-length paxillin gene under the expression of a CMV promoter (Ad-Pxn). Overexpression of paxillin significantly increased cell migration and wound closure (Fig. 3A), with the wound in HSCs overexpressing paxillin almost completely closing by day 6 compared to control HSCs (Fig. 3A). Furthermore, HSCs overexpressing paxillin had increased proliferation (Fig. 3B) and attachment (Fig. 3C). We additionally created an adenovirus with shRNA against paxillin under the expression of a human U6 promoter (Ad-shPxn). Paxillin knockdown led to reduced HSC migration (Fig. 3A), proliferation (Fig. 3B) and attachment (Fig. 3C). The successful knockdown of paxillin (>90%) was confirmed with western blotting (Fig. S1C). Collectively, these observations suggest that paxillin appears to be involved in the regulation of various phenotypes associated with activated HSCs.

### Paxillin increases type I collagen expression

**Fig. 4.**
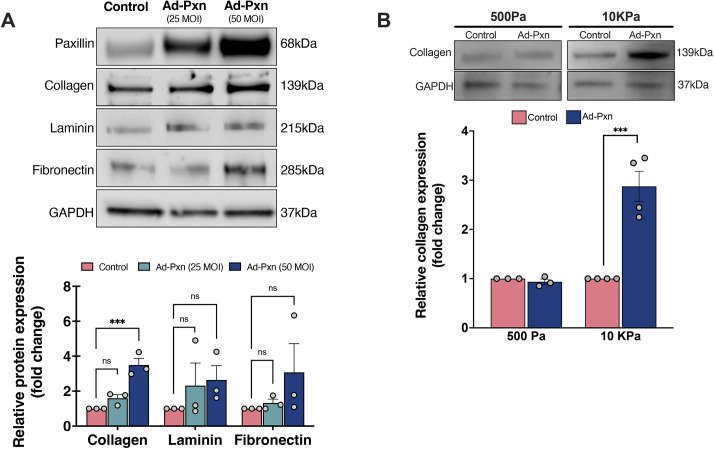
**The effect of paxillin overexpression on ECM production in primary rat HSCs.** (A) HSCs were exposed to different concentrations of Ad-Pxn, and the expression of three different ECM proteins (collagen, laminin, and fibronectin) in cell lysates was measured by immunoblotting as described in the Materials and Methods. Representative immunoblots are shown. Specific bands were scanned and quantified. Data are presented graphically below (*n*=3 biological replicates). (B) Primary rat HSCs were isolated and grown on polyacrylamide hydrogels with low stiffness (500 Pa, mimicking normal livers) and high stiffness (10 kPa, mimicking fibrotic livers), and exposed to Ad-Pxn (50 MOI). Type I collagen was detected by immunoblotting. A representative immunoblot is shown. Specific bands were scanned and quantitated, and data are presented below (*n*=3-4 biological replicates). Error bars represent the mean±s.e.m*.* ***P<0.001; ns, not significant (one-way ANOVA with Tukey multiple comparisons in A, two-tailed unpaired Student's *t*-test in B). Ad, adenovirus; Pxn, paxillin.

Liver fibrosis is characterized by upregulated secretion and deposition of ECM material by HSCs (Koyama and Brenner, 2017). To explore the role of paxillin in HSC fibrogenesis, we overexpressed it in primary rat HSCs using Ad-Pxn and examined the expression of three ECM proteins (type I collagen, laminin and fibronectin) in HSC lysates (Fig. 4A). Paxillin overexpression resulted in a significant increase in type I collagen expression. We also found that the expression of type I collagen, laminin and fibronectin was significantly elevated in conditioned medium from HSCs after paxillin overexpression (Fig. S2). Taken together, these results demonstrate that paxillin positively regulates the expression and secretion of ECM proteins, and imply that paxillin is an important modulator in HSC activation.

### Paxillin-mediated increase in type I collagen production appears to be associated with substrate stiffness

Substrate stiffness dictates the degree of HSC activation and subsequent ECM production (Olsen et al., 2011). Given that paxillin plays a key role in the cellular response to mechanical signaling from the ECM (Jansen et al., 2017), we tested whether matrix stiffness could stimulate paxillin-mediated increases in type I collagen. We synthesized polyacrylamide hydrogels of two different stiffnesses – 500 Pa and 10 kPa – resembling normal and fibrotic livers, respectively (Olsen et al., 2011). When cultured on non-stiff substrates, HSCs overexpressing paxillin did not increase the production of type I collagen (Fig. 4B). In contrast, overexpressing paxillin in HSCs cultured on stiff substrates led to an increase in the expression of type I collagen. Thus, it appears to be that paxillin modulates the increase in collagen production mediated by increased substrate stiffness.

### Paxillin stimulates actin polymerization

**Fig. 5.**
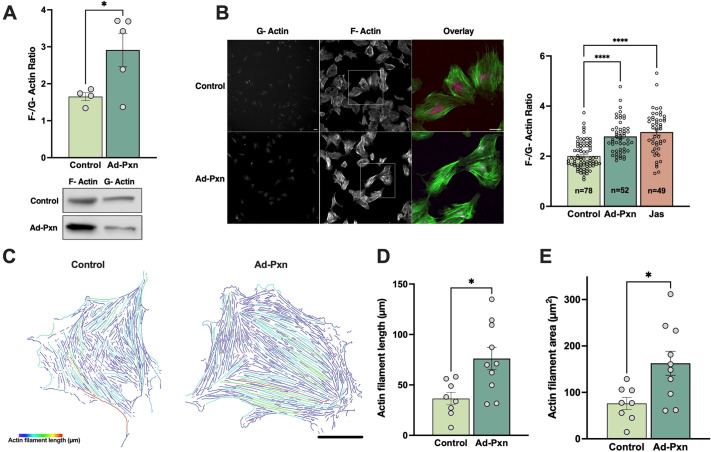
**Paxillin overexpression leads to increased actin polymerization.** (A) HSCs were isolated from normal rat livers and after 2 days exposed to Ad-Pxn for 2 days, switched to 0.5% serum medium for 24 h, and then subjected to F-/G-actin measurement. F- and G-actin isoforms were detected by immunoblotting with actin isoform-specific antibodies and the F-/G-actin ratios were calculated as described in the Materials and Methods. A representative blot is shown below (*n*=4–5 biological replicates). (B) HSCs were grown as in A above, and after incubation in 0.5% serum medium for 24 h, were fixed and stained with phalloidin and DNase I as described in the Materials and Methods. Some cells were exposed to jasplakinolide (100 nmol/l, 1 h prior to fixation) as a positive control for actin polymerization. Scale bar: 50 μm. The F-/G-actin ratio was calculated (shown to the right) using the fluorescence intensity of phalloidin and DNase I, respectively. *n*=78, 52 and 49 for control, Ad-Pxn and Jasplakinolide, respectively. *n* is number of cells analyzed from four biological replicates. (C) The actin cytoskeleton of cells exposed to Ad-Pxn were skeletonized using Imaris software. A representative example is shown. Scale bar: 30 μm. (D) Actin filament length was measured in HSCs as in C (*n*=8–10 cells, each from the biological replicates). (E) HSCs as in C were used to measure actin filament area. *n*=8 cells from three biological replicates for control and 10 cells from three biological replicates for Ad-Pxn. Error bars represent the mean±s.e.m*.* *P<0.05; ****P<0.0001 versus control (two-tailed unpaired Student's *t*-test in A, D and E; one-way ANOVA with Tukey multiple comparisons in B).

Next, we investigated the effect of paxillin overexpression on actin polymerization in HSCs by measuring the ratio of polymerized filamentous actin (F-actin) to that of monomeric globular actin (G-actin). We found a significant increase in the F- to G-actin ratio in HSCs overexpressing paxillin using two different methods (Fig. 5). First, we examined F- and G-actin using immunoblotting (Fig. 5A). In addition, we stained F- and G-actin using phalloidin and DNase I, respectively, and calculated their fluorescence intensity ratio (Fig. 5B). Jasplakinolide, which is known to stimulate actin polymerization, was used as a positive control. Finally, actin cytoskeletons of individual cells were skeletonized (Fig. 5C) as in the Materials and Methods and quantitative analysis of individual actin filaments in HSCs overexpressing paxillin revealed significant increases in both actin filament length (Fig. 5D) and area (Fig. 5E) in HSCs overexpressing paxillin. Taken together, these experiments imply that paxillin might be involved in the changes of the actin cytoskeleton that occur during HSC activation.

### The interaction between paxillin and actin is augmented in activated HSCs

We immunoprecipitated paxillin from quiescent and activated HSCs using anti-paxillin antibody and demonstrated two unique bands upon Coomassie Blue staining of the proteins harvested from activated HSCs (Fig. S3A). Mass spectrometry protein identification revealed these bands to be actin and myosin (predominantly MYH9, but also MYH10 and MYH11), respectively. Furthermore, when paxillin was immunoprecipitated from activated HSCs, actin was found to be associated with paxillin, whereas there appeared to be no association of paxillin and actin in quiescent HSCs (Fig. S3B). As a control, we analyzed samples from activated HSCs using identical conditions, but omitting anti-paxillin antibody or using mouse IgG instead (Fig. S3C,D). Finally, we performed immunoblotting on lysates from activated HSCs immunoprecipitated with anti-paxillin antibody, and identified two actin isoforms [smooth muscle α-actin (ACTA2) and β-actin (ACTB)] that interact with paxillin (Fig. S3E,F). Actin has six different isoforms, each of which performs unique cellular functions; quiescent HSCs express only β- and γ-cytoplasmic actin isoforms, whereas activated HSCs also express abundant smooth muscle α-actin, which forms robust and well-organized actin filaments that contribute to HSC contraction, proliferation, and migration (Hijazi et al., 2022). These data further suggest that the interaction between paxillin and actin is enhanced in activated HSCs.

### Paxillin stimulates type I collagen production via the actin cytoskeleton and the ERK pathway

**Fig. 6.**
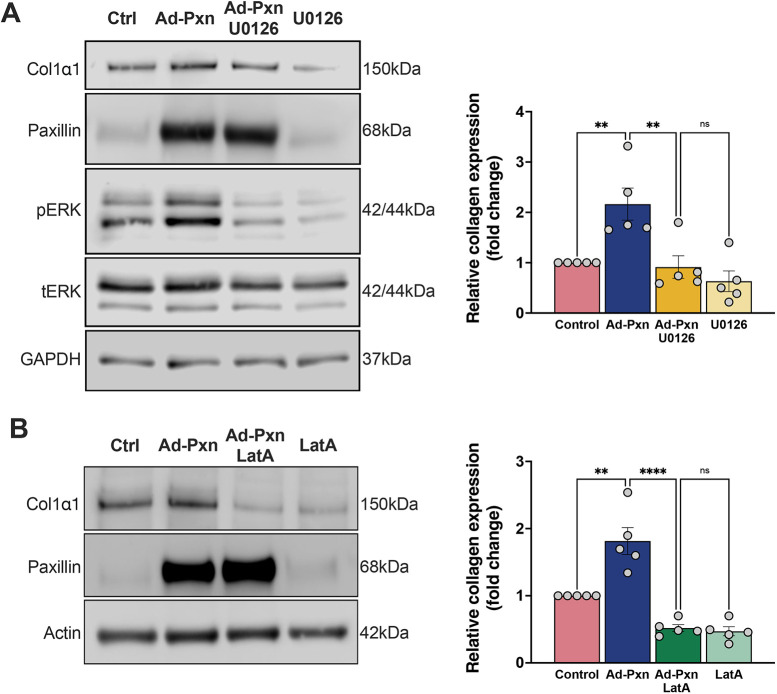
**Paxillin stimulates type I collagen expression via ERK1/2 activation and actin polymerization.** (A) HSCs were isolated and grown for 3 days, exposed to Ad-Pxn for 2 days, and incubated in 0.5% serum medium with or without 10 μM of U0126 (an ERK inhibitor) for 24 h. Cell lysates were harvested and subjected to immunoblotting to detect the indicated proteins. Representative images are shown on the left and specific bands from all experiments were scanned and quantified, and densitometric analysis is shown to the right (*n*=5 biological replicates). (B) HSCs were cultured as in A and incubated in 0.5% serum medium with or without 1 μM of latrunculin A (to inhibit actin polymerization) for 24 h. Cell lysates were harvested and subjected to immunoblotting to detect the indicated proteins. Representative images are shown on the left and specific bands from all experiments were scanned and quantified, and densitometric analysis shown to the right (*n*=5 biological replicates). **P<0.01; ****P<0.0001 ns, not significant (one-way ANOVA with Tukey multiple comparisons). Ad-PXN, adenovirus paxillin; pERK, phoshoERK; tERK, total ERK.

Given that it has been previously established that the ERK pathway plays an integral role in the profibrogenic phenotype of activated HSCs (Foglia et al., 2019), we hypothesized that paxillin might be involved in ERK1/2-dependent fibrogenic signaling. We overexpressed paxillin in activated HSCs and found an increase in ERK1/2 phosphorylation (Fig. 6A, lane 2), with no effect on total ERK. The increase in type I collagen was abrogated in Ad-Pxn HSCs exposed to the ERK1/2 activation inhibitor U0126 (Fig. 6A). These data suggest that the profibrogenic phenotype observed after paxillin overexpression is mediated via the ERK pathway.

Because of the close interaction between paxillin and actin, we further postulated that paxillin might stimulate the actin-ERK-collagen pathway. Inhibition of actin polymerization in HSCs overexpressing paxillin using latrunculin A (LatA) led to a decrease in the increase of type I collagen caused by paxillin alone (Fig. 6B). Thus, it appears to be that paxillin increases the production of type I collagen through the actin-ERK pathway.

### Paxillin stimulates liver fibrosis *in vivo*

**Fig. 7.**
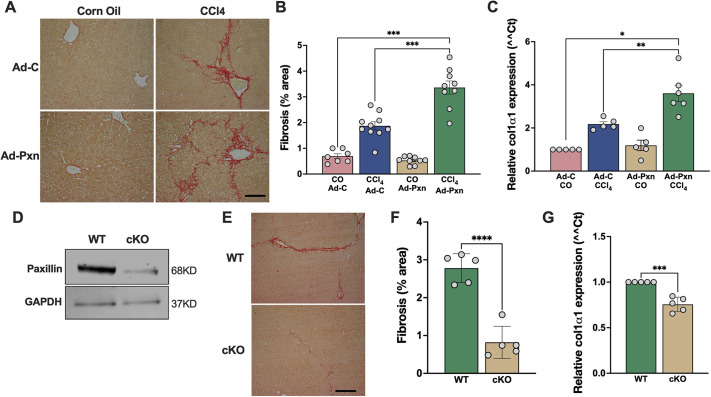
**Paxillin stimulates liver fibrosis *in vivo*.** (A) C57/BL6 mice were divided into four groups and were given CCl_4_ (or corn oil as a control) and/or injected with 2×10^11^ adenoviral (Ad) particles Ad-Pxn [or GFP containing adenovirus (Ad-C) as a control]. At 6 days after the final CCl_4_ (or corn oil) dose, whole-liver tissues were harvested and subjected to Picrosirius Red staining (to determine fibrotic area) as described in the Materials and Methods. Representative images are shown. Scale bar: 100 μm. (B) Histomorphometric analysis of collagen content (red) in random sections was performed as described in the Materials and Methods and quantitative data are presented graphically (*n*=corn oil+Ad-C, 7 mice; CCl4+Ad-C, 10 mice; corn oil+Ad-Pxn, 9 mice; CCl4+Ad-Pxn, 9 mice). (C) Total RNA was isolated from whole-liver tissues and subjected to qPCR to detect *Col1a1* (col1α1) expression as described in the Materials and Methods (*n*=5 biological replicates). (D) Western blot showing paxillin expression in HSCs isolated from paxillin WT and cKO mice (representative of *n*=3 repeats). (E) Liver injury was induced in paxillin^+/+^/MYH11-Cre+ (WT) and paxillin^Fl/Fl^/MYH11-Cre+ (cKO) mice. Whole-liver tissues were harvested and subjected to Picrosirius Red staining as described in the Materials and Methods. Representative images are shown. Scale bar: 100 μm. (F) Histomorphometric analysis of collagen content (red) in random sections was performed described in the Materials and Methods and quantitative data are presented graphically (*n*=5 WT biological replicates, 5 cKO biological replicates). (G) Total RNA was isolated from whole-liver tissues and subjected to qPCR to detect *Col1a1* expression as above (*n*=5 biological replicates). *P<0.05; **P<0.01; ***P<0.001; ****P≤0.0001 (one-way ANOVA with Tukey multiple comparisons in B and C, two-tailed unpaired Student's *t*-test in F and G). Ad, adenovirus; cKO, conditional knockout; c, control, CO, corn oil; Pxn, paxillin.

To further explore the role of paxillin *in vivo*, we examined the effect of overexpression of paxillin using an adenovirus expressing wild-type (WT) paxillin in a CCl_4_-induced liver fibrogenesis model. As demonstrated previously, adenovirus efficiently infects hepatic non-parenchymal liver cells, including HSCs, and leads to robust gene expression (Yu et al., 2002). Adenoviral-mediated delivery of paxillin led to increased paxillin (and GFP in control) expression in the liver (Fig. S4). Measurement of fibrosis in whole liver tissue was assessed using Picrosirius Red staining of collagen, as described in the Materials and Methods: mice that received Ad-Pxn developed significantly more fibrosis than control mice (Fig. 7A). Quantitative morphometric analysis indicated mice overexpressing paxillin had a 71% increase in fibrosis area versus control (Fig. 7B, lanes 2 and 4). Uninjured, normal mice in which paxillin was overexpressed did not have elevated levels of fibrosis (Fig. 7B, lanes 1 and 3). Quantitative (q)PCR analysis on whole liver RNA also showed that paxillin expression led to increased mRNA expression of type I collagen compared with mice receiving control adenovirus (Ad-C) (Fig. 7C).

We also examined the effect of paxillin knockdown *in vivo* on fibrogenesis by utilizing an inducible mouse model to delete paxillin specifically in HSC as described in the Materials and Methods. Depletion of paxillin was verified by isolating HSCs from WT and conditional knockout (cKO) mice and examining the levels of paxillin (Fig. 7D). Following CCl_4_ administration and ensuing liver injury, with induction of paxillin knockdown, quantitative morphometric analysis revealed a significant decrease in fibrosis in cKO mice (Fig. 7E,F). Furthermore, qPCR analysis of whole liver RNA also demonstrated a reduction in *Col1a1* expression in cKO mice compared to WT mice (Fig. 7G). Collectively, these results imply that paxillin is a key regulator in the fibrogenic response that underlies liver fibrosis.

## DISCUSSION

**Fig. 8.**
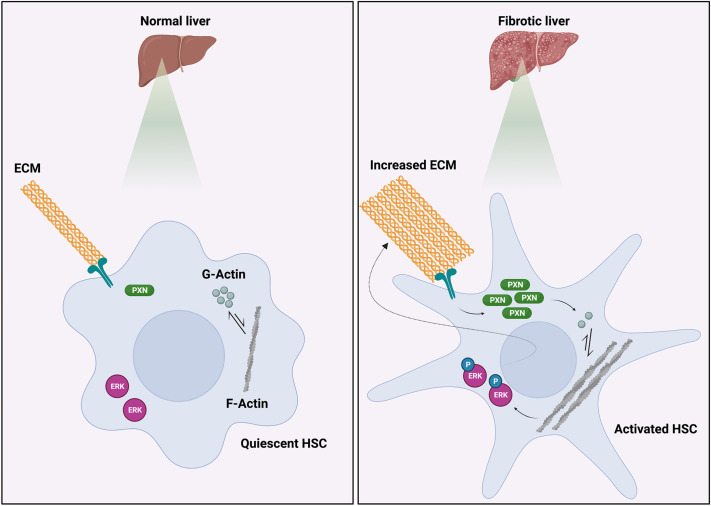
**Schematic diagram of the working mechanism for paxillin biology in HSCs and liver fibrosis.** In fibrotic livers, elevated ECM stiffness triggers the upregulation of paxillin production, leading to enhanced actin polymerization and subsequent synthesis of type I collagen through the activation of the ERK pathway. ERK, ERK1 and/or ERK2; PXN, paxillin. Created with Biorender.com.

In this study, we have demonstrated that paxillin is upregulated in HSCs during liver injury and fibrogenesis. Furthermore, we have shown that paxillin has important functional effects on isolated HSCs, which in turn are crucial in the biology of fibrosis. We also demonstrated that paxillin has important effects on fibrogenesis *in vivo*. Previous studies from our laboratory suggest that the ERK pathway is implicated in the actin cytoskeleton-mediated regulation of type I collagen (Rockey et al., 2013). The mechanism by which paxillin appears to exert its functional effects is via stimulation of the actin cytoskeleton and subsequent activation of the ERK-collagen pathway (Fig. 8). Taken together, these findings highlight paxillin as a novel potential therapeutic target for liver fibrosis.

To our knowledge, this is the first study to examine the role of paxillin in HSC activation and liver fibrosis. Although these findings are novel, they are consistent with a previous study showing that focal adhesion kinase (FAK, also known as PTK2, another important FA protein) promotes HSC activation *in vitro* and liver fibrosis *in vivo* (Zhao et al., 2017). Given that paxillin and FAK closely interact and are recruited early during FA formation, our study raises the possibility of a connection between these two FAs, and further emphasizes the important role that FA proteins appear to have in hepatic fibrogenesis.

We have demonstrated that paxillin is upregulated in activated HSCs. The increase in paxillin expression might be due to multiple factors. For one, paxillin-containing focal adhesions have been shown to increase in number and density with increased substrate stiffness (Oakes et al., 2018). This is consistent with an experiment we performed in which we grew HSCs on synthesized matrices with different stiffnesses and showed that the greater the stiffness, the higher the paxillin expression (data not shown). These data are also consistent with our other findings (Fig. 1) because it is well known that both *in vitro* and *in vivo* activated HSCs produce ECM that serves as a substrate for HSCs (Wells, 2008; Tsuchida and Friedman, 2017), thus stimulating HSC activation in an autocrine process.

Given that paxillin is one of the downstream regulators of mechanotransduction and has the ability to respond to changes in the physical microenvironment of the cell, such as increased stiffness (Jansen et al., 2017; Kang, 2020), we asked whether its ability to increase ECM production is stiffness-related. In other words, does paxillin initiate fibrogenesis or lead to its progression? Using hydrogels of different stiffnesses, we found that paxillin overexpression increased type I collagen only on stiff matrices. This raises the possibility that paxillin potentiates liver fibrosis rather than initiates it. To gain a better understanding of the involvement of paxillin in fibrogenesis, it would be intriguing to examine alternative hypotheses, such as the notion that paxillin plays a role in initiating liver fibrosis.

In this study, we have focused on the canonical form of paxillin (α isoform). However, alternative splicing of paxillin pre-mRNA produces three other isoforms (β, γ and δ) that exhibit different expression patterns and have different functions (López-Colomé et al., 2017). Interestingly, it has been proposed that paxillin-δ serves as a naturally occurring functional antagonist of canonical paxillin signaling during processes associated with cell migration (Tumbarello et al., 2005). Our data thus indicate that the α-isoform of paxillin is crucial in HSC biology, and whether the other isoforms of paxillin are required for HSC function remains unclear.

Studies from our laboratory and others have demonstrated that the development of a robust actin cytoskeleton is one of the key molecular mechanisms involved in the upregulation of type I collagen production in activated HSCs (Rockey et al., 2019, 2013; Shi and Rockey, 2017). Paxillin is known to tether actin filaments to focal adhesions and to be involved in actin cytoskeleton polymerization and remodeling (López-Colomé et al., 2017; Turner, 2000; Rembold et al., 2007; Turner, 1994). Here, we also show a correlation between paxillin expression and actin polymerization in HSCs, consistent with similar findings in other cell types (Rembold et al., 2007; Zhang et al., 2007). Interestingly, we found that paxillin appears to bind to both β-actin and smooth muscle α-actin (Fig. S3E,F), pointing to interaction of paxillin with conserved elements of actin isoforms. Given that the expression of the actin isoforms varies from one cell type to another, exploration of the interaction between paxillin and specific actin isoforms will likely be of substantial interest.

One of our previous studies showed that the ERK pathway is involved in the actin cytoskeleton-mediated regulation of type I collagen production through phosphorylating downstream effectors (c-FOS and c-JUN) which bind to a putative AP-1-binding site in the type I collagen gene promoter (Rockey et al., 2013). We also previously demonstrated that the interruption of actin polymerization with the actin disruptor latrunculin B, or with the Rho kinase inhibitor CCG-203971, blocked ERK1/2 phosphorylation as well as type I collagen expression (Rockey et al., 2019; Shi et al., 2020; Shi and Rockey, 2017). Here, we further demonstrate that inhibiting ERK phosphorylation using U0126 abrogates paxillin-induced type I collagen expression. These data suggest that paxillin participates in the regulation of type I collagen expression via the actin cytoskeleton-ERK pathway.

Our experiments showed that paxillin overexpression increased HSC migration, proliferation and attachment. Each of these phenotypes has complex underlying molecular mechanisms and examining all of them is beyond the scope of this study. Similar to our findings, a previous study has reported that paxillin-null fibroblasts exhibit reduced migration, and paxillin overexpression has been shown to promote migration in various cell types (Hagel et al., 2002). It does so by coordinating the spatiotemporal activation of the Rho family of GTPases and by regulating focal adhesion turnover (López-Colomé et al., 2017). Paxillin has also been shown to increase the proliferation of HeLa, COS-7 and prostate cancer cells. Possible mechanisms for increased proliferation include suppressing *H19* gene expression which promotes *Igf2* expression (Dong et al., 2009) and potentiating steroid hormone receptor activities (Kasai et al., 2003). As for attachment, which is part of cell migration, previous reports have shown that paxillin is one of the first proteins to localize to sites of adhesion and that its knockdown decreases attachment to substrates (Brown and Turner, 2004; Bukahrova et al., 2005). In this manuscript, our primary emphasis was on investigating the connection between paxillin and collagen production, which represents one aspect of activation. We believe that understanding the role of paxillin on other phenotypes of HSC activation is crucial for gaining a complete understanding of its role in liver fibrosis.

In summary, we have demonstrated a novel role for paxillin in HSC activation and liver fibrosis. Not only does this study shed new light on the cell biology of paxillin and fibrogenic effector cells, but it also highlights paxillin as a potential target for future therapeutic approaches in liver fibrosis.

## MATERIALS AND METHODS

### Cell isolation and culture

Male Sprague-Dawley (SD) rats (500–600 g) were purchased from Charles River Laboratories. All animals were housed in the animal facility at the Medical University of South Carolina (MUSC) and received care following NIH and IACUC guidelines. All animal studies met NIH guidelines for ethical animal care and experimentation, and were approved by the MUSC IACUC. Primary rat HSCs were isolated by *in situ* enzymatic digestion as previously described (Shi and Rockey, 2010) and were grown in 199OR standard medium containing 10% calf serum, 10% horse serum, and an antibiotic-antimycotic solution (cat. number 15240096, Thermo Fisher Scientific). Cell purity (>98%) was assessed by determining the intrinsic vitamin A auto-fluorescence. In experiments utilizing adenovirus, rat HSCs were grown in standard medium and exposed to adenovirus for a further 2 days. Cells were subsequently incubated in medium as above but with 0.5% serum for 24 h before analysis, unless otherwise indicated.

### Animal models of liver injury

Liver injury and fibrosis were induced in rats by repetitive intragastric administration of carbon tetrachloride (CCl_4_) or by bile duct ligation (BDL). CCl_4_ (319961, Sigma-Aldrich) was administered at 1 ml/kg body weight mixed with corn oil (CO) at a 1:1 ratio, once a week for 6 weeks, as previously described (Shi and Rockey, 2017). Controls received corn oil on the same schedule as experimental animals. At 5 days after the last dose of CCl_4_, *in vivo* activated HSCs were isolated as described above. For the BDL model, laparotomy under anesthesia was performed to isolate and ligate the common bile duct as described previously (Liu et al., 2005). Control animals underwent sham laparotomy without BDL. HSCs were isolated 14 days after surgery.

To examine the role of paxillin *in vivo*, we examined both gain and loss of paxillin (via overexpression using adenovirus, and deletion using a conditional knockout, respectively). In overexpression experiments, 35 male mice (C57BL/6, 8–9 weeks old, purchased from The Jackson Laboratory, Bar Harbor, ME, USA) were divided into four groups (corn oil+Ad-C, 7 mice; CCl_4_+Ad-C, 10 mice; corn oil+Ad-Pxn, 9 mice; CCl_4_+Ad-Pxn, 9 mice). Liver injury was induced by administering CCl_4_ (1 ml/kg body weight, mixed with corn oil at a 1:1 ratio) via gastric gavage once a week for 4 weeks. A PureVirus™ Adenovirus Purification Kit (VPK-5112) was used to purify the adenoviruses according to the manufacturer's instructions (Cell Biolabs, San Diego, CA, USA). Purified adenovirus was injected via the tail vein (2×10^11^ viral particles of Ad-C or Ad-Pxn per mouse) at 2 days after the third corn oil or CCl_4_ dose. Livers were harvested 6 days after the fourth corn oil or CCl_4_ dose. A sample size calculation for the *in vivo* experiments determined that, with an estimated effect size of 1.7, five animals per experimental group provides 80% power to detect differences.

To create an HSC-specific conditional paxillin knockout mouse, we crossbred a paxillin loxP floxed (Pxn^Fl/Fl^) mouse (129/Sv strain, gift of Dr Christopher Turner, SUNY Upstate Medical University, USA) with a MYH11-CreERT2 transgenic mouse (C57/BL6 strain, strain #019079), purchased directly from the Jackson Laboratory to generate mixed background (129/Sv-C57/BL6) Pxn^Fl/Fl^/MYH11-Cre+ and Pxn^+/+^/ MYH11-Cre+ male mice. In these mice, Cre recombinase expression causes conditional deletion of paxillin in activated HSCs following liver injury given that we have demonstrated that smooth muscle myosin heavy chain (SMMHC, encoded by *Myh11*) expression is upregulated during HSC activation (Shi and Rockey, 2010). The paxillin^+/+^/MYH11-Cre+ mouse is referred to as the WT and the paxillin^Fl/Fl^/MYH11-Cre+ is referred to as the cKO. Control mice were age- and sex-matched littermates.

Mice were genotyped with DNA obtained from tail digest with the following primers: forward: 5′-GTTTGGGGCTGGACTCTACC-3′ and reverse 5′-TACAGCTAAGGCATGTAGAG-3′. The resulting PCR product yielded a band at 347 bp for the WT paxillin allele and a band at 443 bp for the floxed paxillin allele. Liver injury was induced in 5 WT and 5 KO mice by administering CCl_4_ (1 ml/kg body weight, mixed with corn oil at a 1:1 ratio) via gastric gavage once a week for 4 weeks. Cre recombinase was induced by a 5-day treatment of intraperitoneal tamoxifen mixed with corn oil (intraperitoneal injections at a dose of 75 mg/kg body weight, cat. number T5648, Sigma-Aldrich; with the first dose of tamoxifen administered 3 days after the second dose of CCl_4_). Livers were harvested 6 days after the fourth corn oil or CCl_4_ dose. Primary HSCs were isolated from WT and cKO mice to validate the reduction of paxillin expression in HSCs.

Only mice exhibiting normal behavior and growth were included in experiments. Normal behavior and growth were assessed based on criteria such as weight gain, activity levels and overall behavior. Prior to experiments, no pre-established exclusion or inclusion criteria were set.

### Actin polymerization assay

To determine the relative proportions of cellular filamentous actin (F-actin) and free globular actin (G-actin), we used an F/G-actin *in vivo* assay kit (cat. number BK037; Cytoskeleton) according to the manufacturer's instructions. Briefly, cells were harvested at 25°C using a lysis and F-actin stabilization buffer containing ATP. Lysates were incubated at 37°C for 10 min and cleared from unbroken cells and debris by centrifuging at 350 ***g*** for 5 min. F-actin was then separated from G-actin by centrifuging 100 μl of lysates at 100,000 ***g*** for 60 min at 37°C. The pellet containing F-actin was resuspended with 100 μl of F-actin depolymerization buffer. The samples were kept on ice for 60 min and were mixed by pipetting every 15 min to help pellet resuspension. Finally, 15 μl of each sample from the depolymerized actin pellet (F-actin) and supernatant (G-actin) were subjected to immunoblotting using anti-actin antibody. Images were analyzed by FIJI/ImageJ and the results were presented as the ratio of F-actin to G-actin.

To determine the effect of paxillin on actin polymerization, cells were grown on glass coverslips and co-stained with Alexa Fluor 594–DNase I (cat. number D12372, Thermo Fisher Scientific) at 9 µg/ml and Alexa Fluor 488–phalloidin (cat. number A12379, Thermo Fisher Scientific) at a 1:250 dilution for 30 min. Jasplakinolide (cat. number 42012750UG, Thermo Fisher Scientific) was added at 100 nmol/l to cells 1 h prior to fixation as a positive control for actin polymerization. Images were taken with an Olympus Fluoview Fv10i laser scanning confocal microscope (Olympus, Tokyo, Japan) using Fluoview software (FV10i-SW, Olympus Europe, Hamburg, Germany) with a UPLSAP 10× objective and analyzed using FIJI/ImageJ. Fluorescence intensities of DNase I and phalloidin were calculated, and the ratio of the value for phalloidin was divided by that for DNase to calculate F-/G-actin ratios.

### Quantitative analysis of actin filaments

WT and paxillin-overexpressing HSCs were labeled with phalloidin as described below. *Z*-stack images of individual cells were taken using a 60× objective lens on the Olympus Fluoview Fv10i (Olympus) laser scanning confocal microscope. Frame size was set to 1024×1024 pixels. *Z*-stacks were then opened in the Imaris (Bitplane) software and subjected to further analysis. A surface was created using a smoothing value of 1.5 μm. The thickness of the smallest actin filament was then measured and set as the number to be subtracted from the background. Smaller spots that do not correspond to actin filaments were filtered out. The surface was then masked with the original channel with voxels outside the surface set to 0. We then skeletonized the actin cytoskeleton by adding a filament object and processed the masked surface using the ‘threshold (loop)’ algorithm. We then chose a value of 6 for the ‘minimal ratio of branched length to trunk radius’ and proceeded without selecting the ‘find dendrite branching point’. Further deletion of smaller blebs was performed on the skeletonized image. The actin filaments were then color-coded based on filament length. Data were analyzed and graphs were generated using GraphPad Prism version 9.1.0 for macOS (GraphPad Software, San Diego, CA, USA).

### Quantitative analysis of focal adhesions

WT and paxillin-overexpressing HSCs were labeled with anti-paxillin antibody (cat. number 610051, BD Biosciences, Franklin Lakes, NJ) at a dilution of 1:200. *Z*-stack images of individual cells were taken using a 60× objective lens on an Olympus Fluoview Fv10i laser scanning confocal microscope, and quantitative analysis of focal adhesions was performed as described previously (Horzum et al., 2014). In brief, images were converted into 16-bit and a *Z-*projection with maximum intensity was performed. Subsequently, the following commands were executed using FIJI software: ‘subtract background with a rolling ball radius of 50 pixels’, enhance contrast by running CLACHE plug-in with block size=19, histogram bins=256, and maximum slope=6’, ‘minimize background by applying mathematical exponential (exp)’, and ‘adjust brightness and contrast automatically’. Then, a Mexican Hat filter was applied (http://bigwww.epfl.ch/sage/soft/LoG3D) with a radius of 5. Next, images were converted to binary using the threshold command and finally, the ‘analyze particles’ command was executed and quantitative statistics were collected (e.g. size, area, number of focal adhesions, and % area occupied by focal adhesions).

### Adenovirus generation and infection

For adenovirus-mediated expression of paxillin, full-length paxillin cDNA (GenBank accession number: BC081984.1) was cloned from rat activated HSCs (forward primer with KpnI site: 5′-TACTGGTACCGATGGACGACCTCGACGCCCTGCTG-3′; reverse primer with XhoI site: 5′-CGCCCTCGAGCTAACAGAAGAGCTTCAGGAAGCAGC-3′). Following restriction digestion with KpnI and XhoI, the purified cDNA fragments were inserted into the KpnI and XhoI sites of a pcDNA3.1-flag plasmid vector as previously described (Shi et al., 2020). Then, the FLAG–paxillin expression cassette was released by HindIII and XhoI digestion and inserted into the HindIII-XhoI sites of a pDC316pCMV shuttle vector (Microbix Biosystems Inc., Toronto, Canada) which was co-transfected with pBGHloxΔE1,3-cre viral plasmid (Microbix, Toronto) in 293HEK cells to generate adenovirus-FLAG-paxillin (Ad-Pxn). HEK 293 (CRL-1573™) cells were obtained from the ATCC (Manassas, VA, USA). The cells are routinely tested for mycoplasma contamination and visually inspected for morphology and signs of bacterial contamination. The control adenovirus with GFP expression (Ad-C) was used as described previously (Shi et al., 2020). For the knockdown virus (Ad-shPxn), a paxillin shRNA (sense, 5′-CTAGCGGACAACCCTACTGTGAAATTCAAGAGATTTCACAGTAGGGTTGTCCTTTTTG-3′; anti-sense, 5′-TCGACAAAAAGGACAACCCTACTGTGAAATCTCTTGAATTTCACAGTAGGGTTGTCCG-3′) was ligated between the NheI and SalI sites of a PDC311 shuttle vector (Microbix, Toronto) under the expression of a human U6 (hU6) promoter. The resulting shPaxillin-harboring plasmid was co-transfected with pBGHloxΔE1,3-cre viral plasmid (Microbix, Toronto) into 293HEK cells to generate adenovirus-FLAG-shPaxillin (Ad-shPxn). An empty shuttle vector with hU6 promoter and sh-Scramble was co-transfected with pBGHloxΔE1,3-cre viral plasmid into HEK 293 cells to generate a control adenovirus (Ad-Scramble). Viral screening and purification were performed according to the manufacturer's instructions (Clontech, Mountain View, CA).

### PCR

Total RNA was extracted from primary rat HSCs with TRIzol™ reagent (Thermo Fisher Scientific), and cDNA was generated using qScript™ cDNA SuperMix (QuantaBio, Beverly, MA, USA) according to the manufacturer's instructions. For PCR, amplification of the target sequence(s) was performed using DreamTaq Green PCR Master Mix (K1081, Thermo Fisher Scientific) and samples were directly loaded on a 1.5% agarose gel. Cycling conditions were 25 cycles of 95°C for 30 s, 55°C for 30 s, 72°C for 45 s, and a final extension of 72°C for 10 min. For qPCR, cDNA was amplified using SsoAdvanced Universal SYBR Green Supermix (Bio-Rad) on a Bio-Rad CFX96 Touch real-time PCR detection system with the following cycling conditions: 40 cycles of 95°C for 15 s, 56°C for 30 s, and 72°C for 30 s. Raw Ct data can be found in Table S1. The primers used were as follows: paxillin RT-PCR (forward, 5′-TCCTACCCAACTGGAAACCA-3′ and reverse, 5′-CACTGCGTTCAGCTCCAGTA-3′), paxillin qPCR (forward, 5′-TGGTCCAGAAGGGTTCCACG-3′ and reverse, 5′-ACGAAGGGTGTGAAGCACTCC-3′), and collagen 1α1 RT-PCR and qPCR (forward, 5′-ACTGGTACATCAGCCCAAAC-3′ and reverse, 5′-GGAACCTTCGCTTCCATACTC-3′).

### Immunoblotting

Cultured HSCs were washed twice with ice-cold 1× PBS and lysed using RIPA buffer (150 mM NaCl, 50 mM Tris-HCl pH 8.0, 1% Nonidet P-40, 0.5% sodium deoxycholate and 0.1% SDS) containing protease and phosphatase inhibitors. The PBS solution was prepared by dissolving 9.55 g of powder PBS (cat. no. 56064C, Sigma-Aldrich) in 1 l of deionized distilled water. The solution was then filtered using a 0.22 μm PES bottle-top vacuum filter (431161, Corning, Corning, NY). Lysates were then incubated for 15 min on ice and centrifuged at 17,000 ***g*** for 15 min. The supernatant was harvested, and protein concentration was measured. Equal quantities of proteins (30 μg unless otherwise indicated) were then separated on 4–12% SDS-PAGE gels (Thermo Fisher Scientific) and transferred to nitrocellulose membranes. For experiments involving proteins in cell culture medium, 40 μl of medium per sample was loaded on the gel. Membranes were then blocked [5% BSA in 1× TBS-T (Tris-buffered saline with 0.1% Tween 20) for 1 h] and probed with primary (overnight at 4°C) and secondary (1 h at room temperature) antibodies as indicated. Specific bands were visualized by chemiluminescence (SuperSignal™ West Pico, Thermo Fisher Scientific), images captured by the Syngene G-Box digital imaging system (Chemi XT4; Syngene, Frederick, MD, USA), and specific bands analyzed using FIJI software. Raw densitometric data can be found in Table S1. Primary antibodies used were as follows: anti-paxillin at 1:1000 (cat. no. 610051, BD Biosciences), anti-alpha smooth muscle actin at 1:1000 (cat. no. A2547, Sigma-Aldrich), anti-collagen I at 1:1000 (cat. no. 1310-01, SouthernBiotech, Birmingham, AL, USA), anti-laminin at 1:1000 (cat. no. ab11575, Abcam), anti-fibronectin at 1:1000 (cat. no. ab2413, Abcam), anti-GAPDH at 1:2000 (cat. no. MA5-15738, Thermo Fisher Scientific), anti-phosphoERK at 1:1000 (cat. no. 9101, Cell Signaling Technology), and anti-total ERK at 1:1000 (cat. no. 9102, Cell Signaling Technology).

### Immunocytochemistry

HSCs were grown on glass coverslips (cat. no. 50-192-8951, Thermo Fisher Scientific) coated with 20% 199OR medium for 2 h. Immunofluorescence staining was performed as follows: HSCs were washed with PBS (three times for 3 min each), fixed with 4% paraformaldehyde in PBS for (15 min), permeabilized with 0.5% Triton X-100 in PBS (7 min) and blocked with 3% BSA (30 min). Cells were then incubated with anti-paxillin antibody (cat. no. 610051, BD Biosciences,) at a 1:200 dilution overnight at 4°C, followed by secondary antibody (1 h at room temperature), and phalloidin (30 min at room temperature). Finally, cells were mounted with fluoroshield solution containing DAPI (ab104139, Abcam). Images were captured using the Olympus Fluoview Fv10i laser scanning confocal microscope. The specificity of primary antibodies was confirmed by including the appropriate negative controls (elimination of primary antibody control or secondary antibody). Goat anti-mouse-IgG secondary antibody conjugated to Alexa Fluor™ 555 (cat. number A-21422, Thermo Fisher Scientific) was used at a 1:500 dilution. Images were taken with an Olympus Fluoview Fv10i laser scanning confocal microscope (Olympus, Tokyo, Japan) using Fluoview software (FV10i-SW, Olympus Europe, Hamburg, Germany) with a 60× UPLSAP60xW objective lens (1.2 NA), a 1.0 aperture, and a sampling speed of 2 μs/pixel. System settings were as follows: Alexa Fluor 488: laser wavelength (473 nm), laser transmissivity (35.2%), PMT voltage (586 V), excitation wavelength (473 nm), emission wavelength (520 nm), BF position (490 nm), and BF range (100 nm); DAPI: laser wavelength (405 nm), laser transmissivity (29.6%), PMT voltage (458 V), excitation wavelength (405 nm), emission wavelength (461 nm), BF position (420 nm), and BF range (40 nm); Alexa 555: laser wavelength (559 nm), laser transmissivity (25.9%), PMT voltage (648 V), excitation wavelength (559 nm), emission wavelength (612 nm), BF position (570 nm), and BF range (100 nm); image size: 1024 pixel (212.13 μm)×1024 pixel (212.13 μm), 12 bits/pixel.

### Quantitative morphometry

Whole-mouse liver tissue was fixed in 10% buffered formalin for 2 days and 5 μm paraffin sections were stained with Picrosirius Red and counterstained with Fast Green FCF as described previously (Rockey and Chung, 1996). In brief, after dehydration and staining with saturated picric acid containing 0.1% Sirius Red (cat. no. 09400, Polysciences Inc., Warrington, PA, USA) and 0.1% Fast Green FCF (cat. no. F7252, Sigma-Aldrich), stained sections were mounted with Permount solution (cat. no. SP15-500, Thermo Fisher Scientific) and covered with a glass coverslip. Images were captured with a Nikon Digital Imaging microscope (Nikon, Tokyo, Japan) and for each animal, and ten microscopic fields were randomly selected (slides were numbered or coded in a way that concealed their respective conditions until after the images were taken and analyzed to ensure data were anonymized), the proportionate fibrotic area in each field was measured using NIH ImageJ. Data were analyzed and graphs were generated using GraphPad Prism version 9.1.0 for macOS (GraphPad Software, San Diego, CA).

### Polyacrylamide hydrogel synthesis

Polyacrylamide hydrogels of different stiffness were synthesized as described with minor modifications (Cretu et al., 2010). Gels with stiffnesses of 500 pascals (Pa) and 10 kPa, resembling matrices of normal and fibrotic livers, respectively, were synthesized. In brief, the top coverslips (25 mm round), were autoclaved and siliconized with a 10% Surfasil (cat. number TS-42800, Thermo Fisher Scientific) solution in acetone for 5 min, then rinsed with acetone followed by methanol. The bottom coverslips were autoclaved, incubated with 0.1 M NaOH for 3 min, 3-APTMS (cat. no. 13822-56-5, Sigma-Aldrich) for 30 min, washed with deionized water (three times for 3 min each), incubated with 0.5% glutaraldehyde (cat. no. S25341, Thermo Fisher Scientific) for 30 min, and finally washed again with deionized water. The gels were then synthesized as follows: for the 500 Pa gels, 38 µl of 2% bis-acrylamide, 183 µl of 30% acrylamide and 779 µl of PBS were mixed. For the 10 kPa gels, 141 µl of 2% bis-acrylamide, 286 µl of 30% acrylamide and 779 µl of PBS were mixed. Finally, 10 µl of APS followed by 3 µl TEMED were added to begin the polymerization process. Before the gel has polymerized, 200 μl of the mix was pipetted on each bottom coverslip. The top coverslip was then added on top of the gel until complete polymerization. Finally, the top coverslips were removed, gels were washed with PBS (three times for 3 min each), incubated with 0.025% sulfo-sanpah (cat. no. 803332, Sigma-Aldrich) and exposed to UV for 10 min. The gels were then washed and incubated with 0.1 mg/ml of fibronectin at 4°C overnight. Subsequently, gels were washed, and exposed to UV light for 30 min before cell seeding.

### Immunoprecipitation

Cell lysates were harvested using an IP lysis buffer (150 mM NaCl, 50 mM Tris-HCl pH 8.0, 1% Nonidet P-40 and 0.5% sodium deoxycholate). Equal cell lysate quantities were immunoprecipitated overnight at 4°C with mouse anti-paxillin antibody (1 μl per 50 μg of protein) or normal mouse IgG for controls. Subsequently, protein A/G agarose beads (cat. no. 20421, Thermo Fisher Scientific) were added to the antigen-antibody mixture (15 μl bead slurry per 1 μg of antibody) and samples were incubated for 2 h at room temperature. The flowthrough was collected by gentle centrifugation at 350 ***g*** in a table-top centrifuge for 3 min and antigen-antibody-bead mixtures were washed three times with 0.5 ml IP lysis buffer (RIPA buffer without SDS). Finally, immune complexes were eluted by incubating samples at 95°C for 7 min in 4× LDS sample buffer (cat. no. NP0007, Thermo Fisher Scientific). The supernatant was then collected and subjected to immunoblotting.

### Wound healing assay

Cell migration was measured using a wound healing scratch assay as described previously (Rockey et al., 2019). In brief, HSCs were grown on plastic 12 well plates in standard 20% serum 199OR medium until they formed confluent monolayers. A uniform scratch was then made using a sterile p20 pipette tip. Cell culture media was changed to 199OR plus 0.5% serum medium (to prevent cell proliferation). Wound closure in HSCs was monitored by taking images using a Leica DMIL LED microscope or a Nikon Eclipse TE300. Cell migration was determined by measuring the area of the wounds throughout their healing process using FIJI/ImageJ.

### Cell detachment assay

Cell detachment was assessed as previously described (Simon and Linstedt, 2018). In this method, equal numbers of primary HSCs (5×10^5^ cells/ml) were seeded on 12-well plates and grown for 2 days before the addition of adenoviruses. After 2 daysr, cells were washed twice with PBS and 0.25% Trypsin-EDTA was added for 5 min. The medium containing floating cells was collected, cells were dyed with Trypan Blue at a 1:1 ratio, and dye-excluded cells were counted using a hemocytometer. The remaining adherent cells were fully trypsinized for 10 min and counted as above. Cell detachment was determined follows: (floating cells)/(floating cells+adherent cells)×100.

### Cell proliferation assay

HSCs were isolated and grown in 96-well plates at 5×10^5^ cells/ml (original plating density) in standard 199OR containing 20% serum medium. On day 5 of culture, the medium was changed to 199OR medium containing 0.5% serum. PDGF-BB (10 ng/ml, CYT-501; ProSpec, East Brunswick, NJ, USA) was added as a positive control for proliferation. Cell proliferation was measured by the MTS method (G3582, Promega) according to the manufacturer's instructions. Briefly, 20 μl of the reagent provided in the kit was directly added to culture wells, which were then incubated for 4 h. Cell proliferation was measured by recording the absorbance at 490 nm which directly correlates with the number of living cells in culture. Raw MTT assay data can be found in Table S1.

### Statistical analysis

Statistical analyses were performed with GraphPad Prism Version 9.1.0. Error bars represent the mean±s.e.m. A two-tailed unpaired Student's *t*-test or one-way ANOVA with Tukey multiple comparisons were used to assess the statistically significant differences between groups. *P* values of the following levels: *P<0.05, **P<0.01, ***P<0.001 and ****P≤0.0001 were considered to be statistically significantly different.

For cell-based experiments, cells are obtained from a single rat for a particular experiment, it is considered a biological replicate with an ‘*n*’ value of 1. As such, cells from three individual rats in an experiment represent *n*=3 biological replicates. This provides 80% power for detecting differences between experimental conditions and enables the identification of effect sizes equivalent to 2.1, assuming a two-sided testing approach with an α level of 0.05.

For *in vivo* studies, mice were randomly assigned to different experimental conditions, such as corn oil versus CCl_4_ treatment. Sample size was based on an anticipated 300% increase in collagen levels; an *n* of 4 or greater provides 80% power to detect effect sizes (group differences relative to their standard deviation) of 1.7 to 2.2.

## Supplementary Material

10.1242/joces.261122_sup1 Supplementary information
