# Supplementary material for: Paxillin regulates liver fibrosis via actin polymerization and ERK activation in hepatic stellate cells
Source: J Cell Sci. 2023 Sep 28;136(18):jcs261122. doi: 10.1242/jcs.261122 (PMC10560551; doi:10.1242/jcs.261122)
Supplement: Supplementary information [file joces-136-261122-s1.pdf]

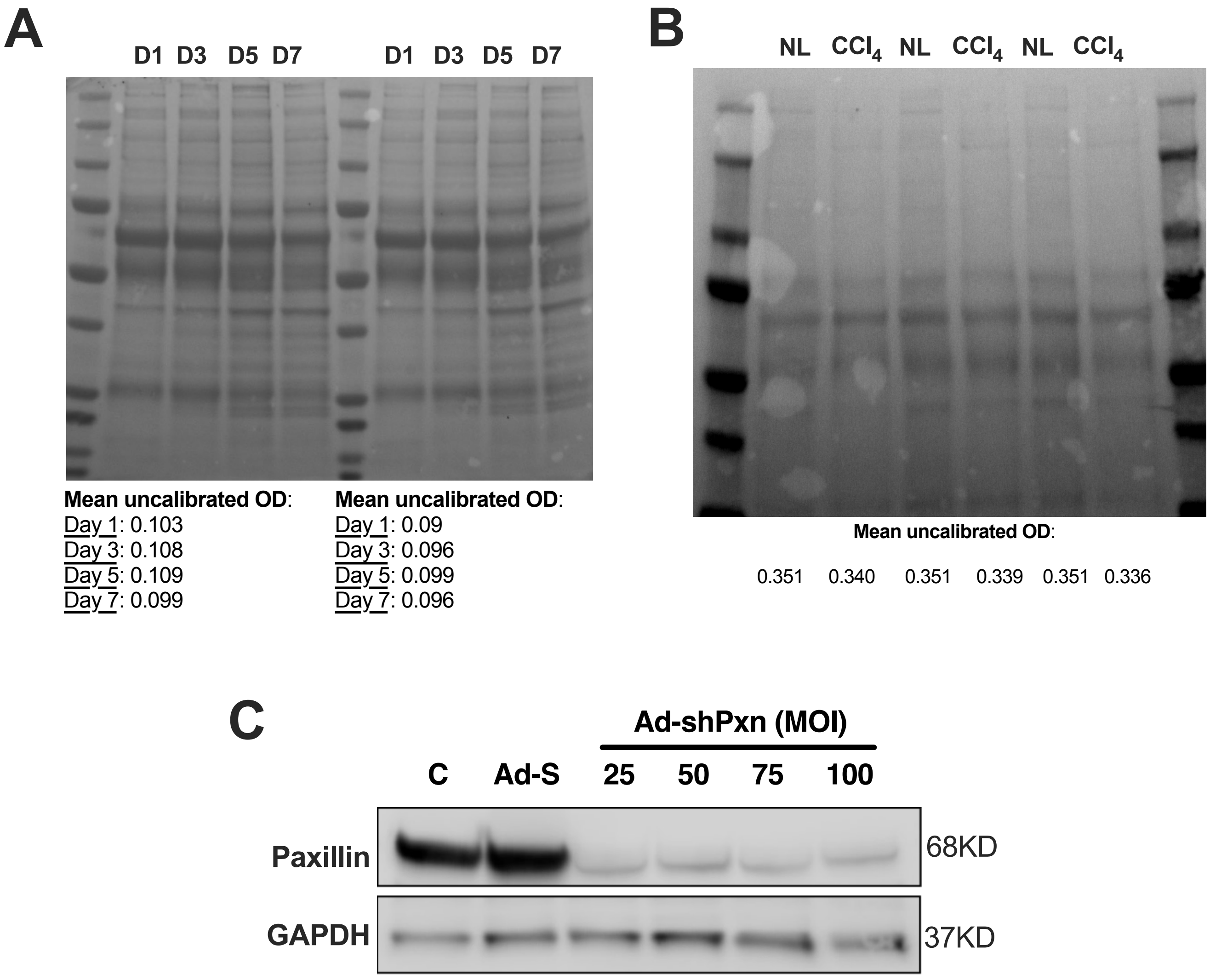

Fig. S1. *Ponceau-S staining corresponding to cell lysates and whole liver lysates in the Western blots shown in Figure 1.* **A:** Shown are molecular weight markers (left lane), and proteins loaded from cell lysates in one representative replicate of culture-activated HSCs (days 1, 3, 5, and 7 – left to right). **B:** Shown are molecular weight markers (left lane), and cell lysates in three replicates of HSCs isolated from normal liver after 1 day in culture (NL) and HSCs isolated from injured liver after 1 day in culture (CCl_4). **C:** Western blot validating the knockdown of paxillin using Ad-shPxn (C: control, Ad-S: control adenovirus containing scrambled shRNA, Ad-shPxn: adenovirus containing shPxn).

Journal of Cell Science • Supplementary information

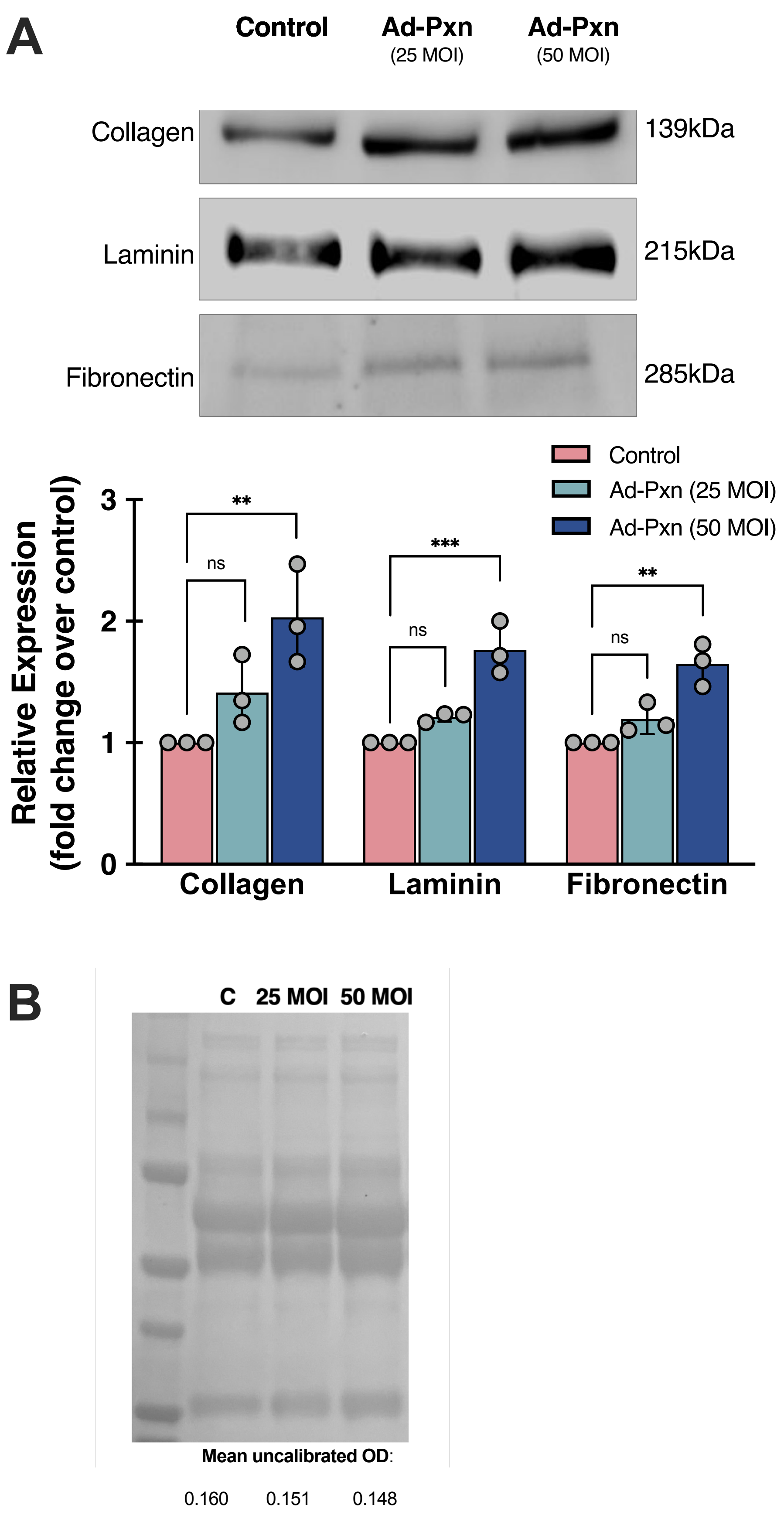

Fig. S2. *Paxillin overexpression causes ECM secretion by HSCs.* ***A:*** Cell culture media was collected from HSCs exposed to 25MOI and 50MOI of Ad-Pxn and the levels of type I collagen, laminin, and soluble fibronectin were measured. Representative images are shown on the top and specific bands from all experiments were scanned, quantitated, and densitometric analysis shown on the bottom. **B**: Ponceau-S stained membrane to show equal loading of protein collected from the culture media of control cells and cells exposed to 25 MOI and 50 MOI of Ad-Pxn is shown.

Journal of Cell Science • Supplementary information

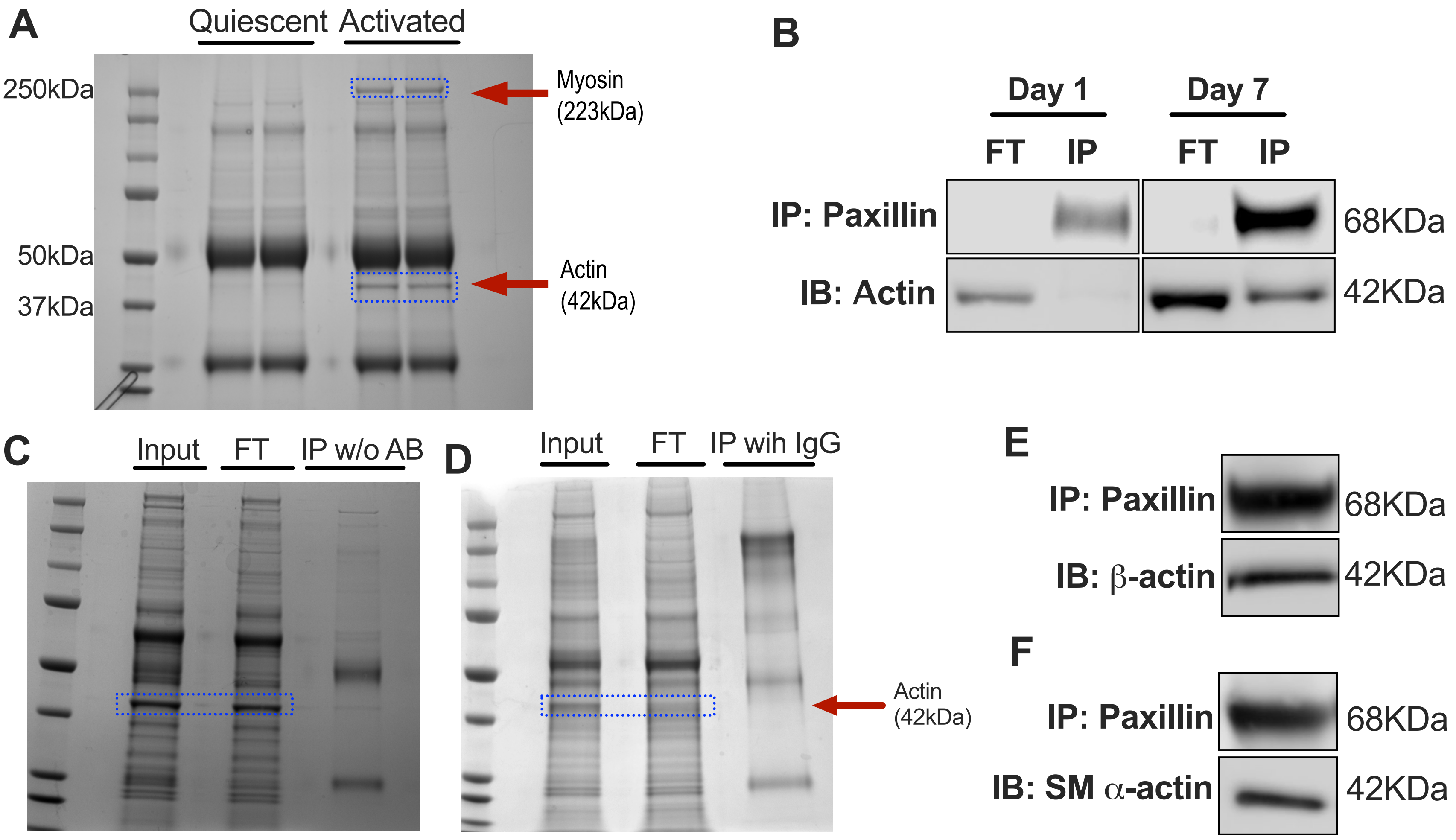

Fig. S3. *Paxillin binding to actin and myosin is increased in activated HSCs.* **A:** HSCs were grown on plastic dishes and proteins were extracted from quiescent HSCs (day 1 in culture) and activated HSCs (day 7 in culture) as in Methods. Cells were harvested and lysates were subjected to immunoprecipitation using protein A/G agarose beads and anti-paxillin antibody as in Methods and gel electrophoresis of bound proteins was performed. A representative gel after Coomassie staining is shown; two bands are depicted by blue dashed boxes. These bands were excised, and mass spectrometry revealed the upper band to be myosin and the lower band to be actin. **B**: Equal amounts of cell lysates were subjected to immunoprecipitation, and eluted proteins were then immunoblotted as in Methods. **C.** Cell lysates were immunoprecipitated as in (A), but not exposed to anti-paxillin antibody. The input, flowthrough (FT), and eluent were subjected to gel electrophoresis. **D.** Cell lysates were immunoprecipitated as in (A) but exposed to mouse IgG antibody. The input, flowthrough (FT), and eluent were subjected to gel electrophoresis. **E-F.** Cell lysates harvested from Day 7 HSCs were subjected to immunoblotting with anti-β-actin (E) or anti-SM muscle α-actin antibody (F), respectively. IP = immunoprecipitation; IB = immunoblotting; FT = flowthrough.

Journal of Cell Science • Supplementary information

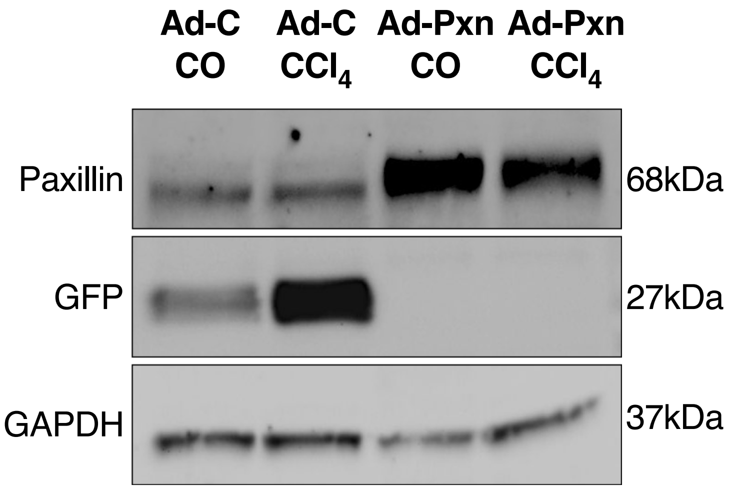

Fig. S4. *Tail vein injection of paxillin-containing adenovirus leads to increased expression of paxillin in normal and injured liver.* C57/BL6 mice were divided into four groups and CCl_4 (or corn oil, CO, as a control) was administered. Adenoviruses containing GFP (Ad-C) as control or paxillin (Ad-Pxn) were injected ($2x10^{11}$ viral particles) two days after the third corn oil or CCl_4 dose. Whole liver specimens were harvested and cell lysates subjected to immunoblotting as in Methods. Ad = adenovirus; C = control; CO = corn oil; GFP = green fluorescent protein, Pxn = paxillin.

Journal of Cell Science • Supplementary information

Table S1. Raw experimental data

Ct values for figure 1A		
	Paxillin	GAPDH
Day 1	28.809	19.425
Day 7	25.166	17.337
Day 1	31.537	21.007
Day 7	24.614	16.921
Day 1	30.400	20.105
Day 7	24.611	16.896

Densitometry data generated for figure 1B			
Day 1	Day 3	Day 5	Day 7
0.058	0.132	0.161	0.212
0.058	0.062	0.099	0.249
0.097	0.168	0.348	0.479
0.058	0.132	0.161	0.212
0.058	0.062	0.099	0.249
0.097	0.168	0.348	0.479

Densitometry data generated for figure 1C		
Control	CCl_4	BDL
0.115	0.651	0.371
0.094	0.53	0.302
0.111	0.841	0.24
0.063	0.761	0.463

MTT assay data for figure 2B			
Control	Ad-Pxn	Ad-shPxn	PDGF
0.26	0.418	0.136	0.481
0.215	0.265	0.114	0.692
0.181	0.47	0.074	0.37
0.317	0.416	0.148	0.46
0.237	0.377	0.108	0.469
0.246	0.419	0.154	0.519

Densitometry data generated for figure 4A (collagen/GAPDH)		
Control	Ad-Pxn (25 MOI)	Ad-Pxn (50 MOI)
0.09	0.164	0.381
0.276	0.489	0.823
0.138	0.163	0.451

Journal of Cell Science • Supplementary information

Densitometry data generated for figure 4B (collagen/GAPDH)	
Control on 10KPa	Ad-Pxn on 10KPa
0.04	0.09
0.09	0.22
0.2	0.67
0.2	0.69

Densitometry data generated for figure 7A (collagen/GAPDH)			
Control	Ad-Pxn	Ad-Pxn + U0126	U0126
0.064	0.106	0.052	0.014
0.085	0.282	0.05	0.033
0.08	0.14	0.05	0.04
0.05	0.12	0.09	0.07
0.072	0.122	0.053	0.047

Densitometry data generated for figure 7B (collagen/acn)			
Control	Ad-Pxn	Ad-Pxn + U0126	U0126
0.055	0.074	0.030	0.016
0.068	0.129	0.032	0.029
0.049	0.124	0.019	0.022
0.066	0.106	0.046	0.046
0.078	0.132	0.038	0.039

Journal of Cell Science • Supplementary information
